# Unprecedented 2015/2016 Indo‐Pacific Heat Transfer Speeds Up Tropical Pacific Heat Recharge

**DOI:** 10.1002/2018GL077106

**Published:** 2018-04-06

**Authors:** Michael Mayer, Magdalena Alonso Balmaseda, Leopold Haimberger

**Affiliations:** ^1^ Department of Meteorology and Geophysics University of Vienna Vienna Austria; ^2^ European Centre for Medium‐Range Weather Forecasts Reading UK

**Keywords:** tropical climate variability, ENSO, Indonesian Throughflow, Indian Ocean, energy budget, reanalysis

## Abstract

El Niño events are characterized by anomalously warm tropical Pacific surface waters and concurrent ocean heat discharge, a precursor of subsequent cold La Niña conditions. Here we show that El Niño 2015/2016 departed from this norm: despite extreme peak surface temperatures, tropical Pacific (30°N–30°S) upper ocean heat content increased by 9.6 ± 1.7 ZJ (1 ZJ = 10^21^ J), in stark contrast to the previous strong El Niño in 1997/1998 (−11.5 ± 2.9 ZJ). Unprecedented reduction of Indonesian Throughflow volume and heat transport played a key role in the anomalous 2015/2016 event. We argue that this anomaly is linked with the previously documented intensified warming and associated rising sea levels in the Indian Ocean during the last decade. Additionally, increased absorption of solar radiation acted to dampen Pacific ocean heat content discharge. These results explain the weak and short‐lived La Niña conditions in 2016/2017 and indicate the need for realistic representation of Indo‐Pacific energy transfers for skillful seasonal‐to‐decadal predictions.

## Key Points

- There was no net ocean heat discharge from the tropical (30° north to 30° south) Pacific during the 2015/2016 El Niño event
- Anomalous Indo‐Pacific heat transfer associated with weak Indonesian Throughflow was a key driver and stronger than surface heat loss
- Reduction of Indonesian Throughflow in 2015/2016 is linked to increased Indian Ocean sea levels associated with preceding enhanced heat uptake

## 1 Introduction

The 2015/2016 El Niño event exhibited peak sea surface temperature (SST) anomalies similar to the record‐breaking 1997/1998 event (Paek et al., 2017). Moreover, it helped to make 2016 the warmest year on record (Potter et al., 2016), likely marking the end of the recent warming hiatus (Hu & Fedorov, 2017).

Besides surface temperatures, there are pronounced perturbations to the energy cycle of the coupled atmosphere‐ocean system associated with El Niño–Southern Oscillation (ENSO), both on a regional and global scale (Loeb et al., 2012; Mayer et al., 2016, 2014; Sun, 2000; Trenberth et al., 2002). Quantifying the energy anomalies associated with ENSO has remained challenging, but recent advances in observational capabilities, most notably in the ocean (Mayer et al., 2014; Von Schuckmann et al., 2016), are making energy diagnostics possible. Recent studies have quantified the energy exchanged between ocean and atmosphere during discharge and recharge phase associated with El Niño and La Niña, respectively (Jin, 1997; Mayer et al., 2014; Trenberth et al., 2014). The largest part of the heat discharged during El Niño is lost through the sea surface via enhanced evaporation and is transported away by the atmosphere from the tropical Pacific region, thereby fueling global teleconnections and rising global mean air temperature (Pan & Oort, 1983; Trenberth et al., 2002).

On average, a small part of the discharged heat is immediately radiated to space in the Pacific subtropics via enhanced outgoing longwave radiation (OLR) (Mayer et al., 2014). However, variability of tropical radiation associated with ENSO crucially depends on the mean state of clouds, which coupled climate models are struggling to reproduce in a realistic manner (Bellenger et al., 2014; Mayer et al., 2016). Moreover, the mean state of clouds is changing as a result of global warming (Clement et al., 2009; Fasullo & Trenberth, 2012) and so will their response to ENSO.

Lateral ocean heat transports have been found to play only a minor role in modulating tropical Pacific average ocean heat content (OHC) away from the equator (Mayer et al., 2014), where there usually occurs pronounced redistribution of heat within the ocean associated with ENSO (Meinen & McPhaden, 2001; Roemmich & Gilson, 2011). One exception is the Indonesian Throughflow (ITF), whose volume and associated heat transports tend to decrease in the course of El Niño events (England & Huang, 2005; Tozuka et al., 2007). However, these ENSO‐related fluctuations of the ITF have been found to play a relatively small role in the average energy cycle of ENSO (Mayer et al., 2014). Apart from interannual variability, the ITF exhibits also pronounced variability on decadal time scales, thereby modulating the warming rate of the Indian Ocean (Lee et al., 2015; Nieves et al., 2015; Ummenhofer et al., 2017), and models predict a general weakening of the ITF associated with global warming (Sen Gupta et al., 2016). In turn, warming of the Indian Ocean can act to modulate Pacific climate change via the atmosphere (Luo et al., 2012).

Here we employ a comprehensive set of atmospheric and oceanic data to characterize the anomalous energy cycle associated with the 2015/2016 El Niño and to contrast it with the 1997/1998 event. Differences between the two events help to identify reasons behind the weak and short‐lived La Niña conditions that followed the 2015/2016 El Niño and help to understand recent weather events that are not expected in years after strong El Niños, like the recurrent strong hurricane seasons in the eastern Pacific during 2016 (Diamond & Schreck, 2017) or the “coastal El Niño” in 2017 bringing torrential rains to Peru and Colombia (Fraser, 2017).

## 2 Data and Methods

### 2.1 Atmospheric Energy Budget

The presented diagnostics are based on the vertically integrated energy budget equations for atmosphere and ocean. The atmospheric energy budget equation reads as

(1) $$F_{S}={\mathrm{Rad}}_{\mathrm{TOA}}-\nabla \cdot F_{A}-\mathrm{AET},$$

where *F*_*S*_ represents net surface energy flux, Rad_TOA_ net radiation at the top‐of‐the‐atmosphere (TOA), ∇ · *F*_*A*_ the divergence of lateral atmospheric energy transports, and AET the rate of atmospheric total energy storage. This work only deals with atmospheric heat transports out of the tropical Pacific region, that is, area integrals of ∇ · *F*_*A*_, which we will term AHT. Variability of AET on interannual time scales is very small and hence is not explicitly discussed. We employ homogenized net radiation at TOA and OLR data from University of Reading (Allan et al., 2014; see supporting information Text S1 for details (Allan et al., 2014; Hersbach et al., 2015)). Absorbed solar radiation (ASR) at TOA is estimated from the difference of Rad_TOA_ and OLR. Atmospheric energy transports are computed using an improved budget formulation (Mayer et al., 2017) and ensuring mass consistency (Mayer & Haimberger, 2012), employing ERA‐Interim (Dee et al., 2011) data from the European Centre for Medium‐Range Forecasts (ECMWF) and the Japanese 55 year reanalysis (JRA‐55; Kobayashi et al., 2015) data from the Japanese Meteorological Agency. Since surface fluxes based on parameterizations are biased and temporally inhomogeneous (Von Schuckmann et al., 2016), we compute *F*_*S*_ as a residual from the right‐hand side of equation (1). For additional diagnostics, total cloud amount is taken from the energy balanced and filled Clouds and the Earth's Radiant Energy system (CERES‐EBAF) version 4.0 (Loeb et al., 2009; Wielicki et al., 1996).

### 2.2 Oceanic Energy Budget

The oceanic energy budget equation reads as

(2) $$F_{S}=\nabla \cdot F_{O}+\text{OHCT},$$

where ∇ · *F*_*O*_ represents the divergence of lateral ocean heat transports (integrated over the full depth of the ocean). OHCT denotes the tendency of OHC. OHC between the surface and depth *d* is computed as

(3) $$\mathrm{OHC}=\rho_{0}c_{p}\int_{0}^{d}\theta \left(z\right)dz,$$

where *ρ*_0_ = 1,026 kg m^−3^ denotes density, *c*_*p*_ = 3,990 J kg^−1^K^−1^ specific heat capacity, and *θ* potential temperature (as a function of depth *z*) of seawater.

For the tropical Pacific (30°N–30°S), which is our main area of interest, we decompose area integrals of ∇ · *F*_O_ into transports across the three‐lateral boundaries of the basin (Gauss's theorem), namely, ITF heat transports (positive westward) and the sum of ocean heat transports across 30°N (OHT_30N_, positive northward) and 30°S (OHT_30S_, positive southward).

OHC fields are available as monthly averages, which are valid in the middle of the respective month. Hence, to obtain unbiased estimates of two‐yearly accumulated OHCT (i.e., two‐yearly OHC changes), which are used widely throughout the paper, we compute differences of two‐monthly averaged OHC. For example, OHCT accumulated from beginning of 1997 to the end of 1999 is computed from the difference of December 1998 to January 1999 and December 1996 to January 1997 averages of OHC. We decompose OHC and OHCT into an upper‐ocean [OHC(0–300 m)] and deep ocean [OHC(300 m − full_depth)] component, since the latter involves much larger uncertainties, especially prior to the Argo (a global array of currently about 3800 temperature and salinity profiling floats; Gould et al., 2004) period, when essentially no in situ measurements existed in the subtropical Pacific below 300 m. This allows us to assign larger uncertainties to the deep ocean OHC, which is relevant for our method to close the energy budget (see below for details). The ocean energy budget for the tropical Pacific (30°N–30°S) then reads as follows:

(4) $$F_{S}=\mathrm{ITF}+{\mathrm{OHT}}_{30N}+{\mathrm{OHT}}_{30S}+\text{OHCT}\left(0-300\;m\right)+\text{OHCT}\left(300\;m-\text{full}\_\text{depth}\right).$$

Ocean heat transports and OHC (referenced to 0°C) are computed from ECMWF's Ocean ReAnalysis System 4 (ORAS4; Balmaseda et al., 2012) and 5 (ORAS5), each consisting of five ensemble members.

ORAS5 is ECMWF's most recent ocean reanalysis and has been developed based on Ocean ReAnalysis Pilot 5 (Tietsche et al., 2014; Zuo et al., 2015), using the same ocean and sea ice model and data assimilation method (see supporting information Text S2 [Good et al., 2013; Titchner & Rayner, 2014] for details).

ORAS5 has a much higher resolution (1/4‐degree horizontal resolution and 75 vertical levels) than ORAS4 (1‐degree horizontal resolution and 42 vertical levels); hence, besides doubling the ensemble size, use of both reanalyses allows to demonstrate the robustness of the results across different model resolutions. Two additional OHC estimates come from a five‐ensemble‐member control run of ORAS5 where no subsurface information has been assimilated and from the entirely in situ based Hadley EN4.2 data (Good et al., 2013). Please note that the latter two OHC estimates are fully independent of each other.

The reanalysis‐based average ITF transports agree very well with independent observations covering a 3 year period (Sprintall et al., 2009). For validation of interannual ITF variability, we resort to estimates based on classic dynamical methods (e.g., Lukas et al., 1996; Wyrtki, 1961). Agreement of reanalysis output with a recent hydrography‐based estimate (Q.‐Y. Liu et al., 2015; see supporting information Text and Figure S3 [Liu et al., 2015; Sprintall et al., 2004, 2009] for results) is very good (*r* ~ 0.8). In addition to ITF transport estimates computed from ORAS4 and ORAS5, we also employ a proxy for ITF volume transports, which relies on sea level anomaly (SLA) difference in the western equatorial Pacific (4°N–6°N; 125°E–135°E) and the eastern Indian Ocean southwest of Sumatra (13°S–8°S; 100°E–118°E; regions indicated in Figure 3a), slightly adopted from Shinoda et al. (2012). Sea level data are taken from ORAS4, ORAS5, and AVISO satellite altimetry. This proxy does not represent a completely independent estimate of ITF volume transports, but SLA in ocean reanalyses is well constrained by satellite observations and hence is deemed less dependent on the dynamics of the underlying ocean model than ITF obtained from the reanalyses.

### 2.3 Forced Budget Closure

One main diagnostic of this study is two‐yearly accumulated budgets integrated over the tropical Pacific area, to compare the energy cycles of the 1997/1998 and 2015/2016 El Niño events. Two‐yearly accumulated budget terms from equations (1) and (2) will not add up to exactly zero, mainly because they are obtained from different largely independent data sets. To close the budget, we choose a variational Lagrange multiplier (*λ*) approach (Bollmeyer & Hense, 2014; Mayer et al., 2014), minimizing the following cost function *J* separately for the 1997/1998 and the 2015/2016 period:

(5) $$J=\frac{{\left({\mathrm{Rad}}_{\mathrm{TOA}}-{\mathrm{Rad}}_{\mathrm{TOA}}\prime \right)}^{2}}{\sigma \prime_{{\mathrm{Rad}}_{\mathrm{TOA}}}^{2}}+\frac{{\left(\mathrm{AHT}-\mathrm{AHT}\prime \right)}^{2}}{\sigma \prime_{\mathrm{AHT}}^{2}}+\frac{{\left(\mathrm{AET}-\mathrm{AET}\prime \right)}^{2}}{\sigma \prime_{\mathrm{AET}}^{2}}+\frac{{\left({\mathrm{OHT}}_{30N}-{\mathrm{OHT}}_{30N}\prime \right)}^{2}}{\sigma \prime_{{\mathrm{OHT}}_{30N}}^{2}}+\frac{{\left({\mathrm{OHT}}_{30S}-{\mathrm{OHT}}_{30S}\prime \right)}^{2}}{\sigma \prime_{{\mathrm{OHT}}_{30S}}^{2}}+\frac{{\left(\mathrm{ITF}-\mathrm{ITF}\prime \right)}^{2}}{\sigma \prime_{\mathrm{ITF}}^{2}}+\frac{{\left({\text{OHCT}}_{0-300\;m}-{\text{OHCT}}_{0-300\;m}\prime \right)}^{2}}{\sigma \prime_{{\text{OHCT}}_{0-300\;m}}^{2}}+\frac{{\left({\text{OHCT}}_{300\;m-\text{full}\_\text{depth}}-{\text{OHCT}}_{300\;m-\text{full}\_\text{depth}}\prime \right)}^{2}}{\sigma \prime_{{\text{OHCT}}_{300\;m-\text{full}\_\text{depth}}}^{2}}+\lambda \left({\mathrm{Rad}}_{\mathrm{TOA}}+\mathrm{AHT}+\mathrm{AET}+{\mathrm{OHT}}_{30N}+{\mathrm{OHT}}_{30S}+\mathrm{ITF}+{\text{OHCT}}_{0-300\;m}+{\text{OHCT}}_{300\;m-\text{full}\_\text{depth}}\right)=\sum_{i}\frac{{\left(F_{i}-F_{i}\prime \right)}^{2}}{\sigma \prime_{i}^{2}}+\lambda \sum_{i}F_{i}\prime$$

Here the primed quantities are the accumulated terms as obtained from the employed data sets. The nonprimed quantities are the adjusted terms. The larger the adjustment of the respective terms (inversely weighted with their uncertainties $\sigma \prime_{i}^{2}$), the larger is the cost *J*. The uncertainties associated with each term are obtained from the spread of the different estimates (10 ocean reanalysis members plus the EN4.2 estimate for OHCT, 10 ocean reanalysis members for ocean heat transports, and 2 atmospheric reanalyses for AHT). Decomposition of OHC into the layers above and below 300 m (see section 2.2) allows to account for larger uncertainties in the deep ocean, especially in the 1990s, when no Argo floats were available. Rad_TOA_ after 2000/2003 is based on CERES‐EBAF data, and hence, its accumulated uncertainty during 2015/2016 is estimated to be 1.0 ZJ (consistent with the global yearly mean uncertainty estimate of 0.24 W m^−2^ provided by Trenberth et al., 2014). The last term in equation (5) represents the hard constraint requiring exact budget closure.

Differentiation of *J* with respect to the primed quantities and *λ* yields eight unknowns in eight equations. The minimum of *J* is found by setting all derivatives to zero. The adjusted quantities are then of the form

(6) $$F_{k}={F_{k}}^{\prime }+\frac{\sigma \prime_{k}^{2}}{\sum_{i}\sigma \prime_{i}^{2}}\sum_{i}{F_{i}}^{\prime },$$

where the last factor represents the sum of all budget terms, that is, the imbalance of our budget estimate. A posteriori uncertainties are estimated from

(7) $$\sigma_{k}^{2}={\left(\frac{1}{\sigma \prime_{k}^{2}}+\frac{1}{\sum_{i}\sigma \prime_{i}^{2}-\sigma \prime_{k}^{2}}\right)}^{-1}.$$

All values denoting two‐yearly changes provided in the following sections are based on this consistent budget estimate. We note, however, that adjustments are generally small compared to the absolute values of the individual terms and are smaller than one standard deviation of the respective uncertainty estimates. A table showing all terms before and after adjustment is provided in supporting information Text and Table S4 (Dee et al., 2011).

### 2.4 Statistics

All diagnostics are based on monthly anomalies with the 1992–2016 mean annual cycle removed. Provided numbers and uncertainties represent means and standard deviation of all available estimates for the respective quantity. If ensemble‐based data sets are involved (as for OHC), means are computed from ensemble means of the available data sets to avoid overweighting of larger ensembles. Uncertainties are always computed from the standard deviation of all available ensemble members. *P* values for Pearson's linear correlation coefficients are based on two‐sided *t* statistics, taking autocorrelation of the involved time series into account.

## 3 Contrasting Pacific Heat Changes During 1997/1998 and 2015/2016

**Figure 1:**
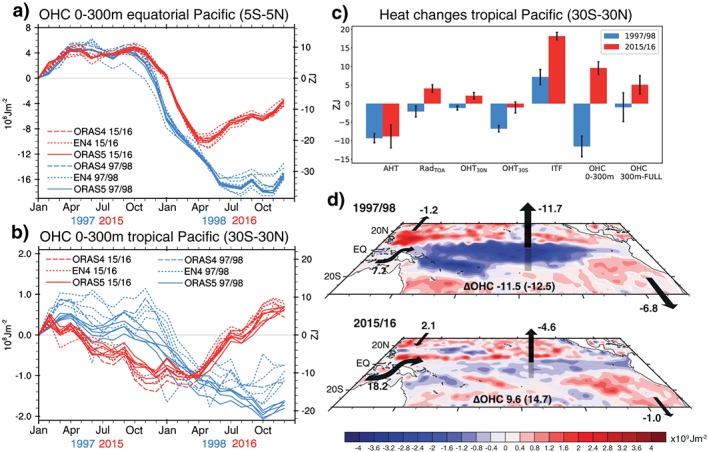
a) Equatorial (5°S–5°N) Pacific ocean heat content (OHC) (0–300 m); (b) tropical (30°N–30°S) Pacific OHC (0–300 m); and (c) accumulated heat budget terms of the tropical Pacific during January 1997 to December 1998 and January 2015 to December 2016: OHC change, contributors to ocean heat transport (Indonesian Throughflow (ITF), OHT_30N_, and OHT_30S_), atmospheric heat transports, and net radiation at top‐of‐the‐atmosphere (Rad_TOA_), including 1*σ* uncertainties. (d) Maps of OHC (0–300 m) changes during January 1997 to December 1998 and January 2015 to December 2016 (based on Ocean Reanalysis System 5, ORAS5). Arrows indicate direction of anomalous heat transports and fluxes across the boundaries of the tropical Pacific (numbers in ZJ). Integrated OHC changes are given for 0–300 m and full‐depth ocean (in brackets). Signs in (c) and (d) are chosen that positive (negative) values indicate a contribution to a warming (cooling) of the Pacific.

Comparison of equatorial (5°S–5°N) Pacific upper (0–300 m) OHC during the two El Niño events shows that discharge lasted almost until the end of 1998, while in 2016 it stopped already by April (Figure 1a). However, the discharge rate (as seen from the similar slope of the curves) during the peak of the two events was similar. The difference in the duration of heat discharge during the two events is consistent with zonally averaged wind stress curl at 5°S and 5°N, respectively, that controls meridional heat transports (Clarke et al., 2007; see supporting information Text and Figure S5 [Paek et al., 2017]) and is likely related to the fact that warm SST anomalies during the 1997/1998 event extended further to the east and persisted for longer (Paek et al., 2017), which favors more persistent wind stress anomalies. In addition, the SST anomaly pattern of the 2015/2016 El Niño had features of Warm Pool events (Paek et al., 2017), which tend to discharge heat from the equatorial band less effectively because of compensating meridional heat transports west and east of the heat content anomaly (Kug et al., 2009). Since a large fraction of equatorial ocean heat discharge represents merely a spatial redistribution of warm waters without a net energetic effect (Meinen & McPhaden, 2001), we will consider the energetics of the full tropical Pacific (30°S–30°N) in the following. This relatively wide definition allows to account for net radiative effects which are strong in the subtropics (Mayer et al., 2014) and to minimize the effect of heat redistribution within the Pacific Ocean.

A striking feature of the 2015/2016 El Niño becomes apparent when considering tropical Pacific (30°S–30°N) upper OHC. At the end of 2016, it was higher by 9.6 ± 1.7 ZJ (Figure 1b) than in early 2015. This is in stark contrast to the 1997/1998 event, which discharged −11.5 ± 3.9 ZJ over a 2 year period, and to statistical analyses generally finding a decrease of tropical Pacific OHC during El Niño events (Mayer et al., 2014, 2016; Trenberth et al., 2014). A forced model run without data assimilation confirms the strong OHC discharge during 1997/1998, which rules out the possibility that the difference between the two events is an artifact of the changing observing system (see supporting information Figure S6).

To identify the drivers of the apparent discrepancy in OHC discharge during 1997/1998 and 2015/2016, we investigate the 2‐year evolution of the relevant energy budget terms for these periods, displayed in Figure 1c. Of all terms, discrepancies in tropical Pacific Ocean heat export (sum of ITF and poleward heat transports) accounts for the by far largest part (74%) of the differences in tropical Pacific total OHC loss, with the main contribution from changes in the ITF, which alone led to an anomalous warming of the tropical Pacific of 18.2 ± 1.0 ZJ during 2015/2016 (see supporting information Figure S7 for accumulated heat transports through the three oceanic gateways). Another remarkable result from Figure 1c is that accumulated net radiation at the TOA was positive during 2015/2016; that is, ASR outweighed OLR anomalies over the tropical Pacific. This is in contrast to the 1997/1998 El Niño, which exhibited a net energy loss to space. While atmospheric heat transports were similar for the two events, the net radiation input at the TOA during 2015/2016 projected onto net surface energy flux anomalies out of the ocean and thereby acted to weaken the negative air‐sea flux feedback. Consequently, net surface heat loss was weaker during 2015/2016 than during 1997/1998.

Maps reveal that over large areas, tropical Pacific upper OHC was far lower following the 1997/1998 El Niño than the 2015/2016 El Niño (Figure 1d). The largest differences are located along the equator and South Pacific convergence zone, indicating that during 1997/1998 more warm water was either transported away from the equator or lost to the atmosphere than during the more recent event. In the following sections we investigate the nature of the different energetics in detail.

## 4 Unprecedented Reduction of the ITF Heat Transport

**Figure 2:**
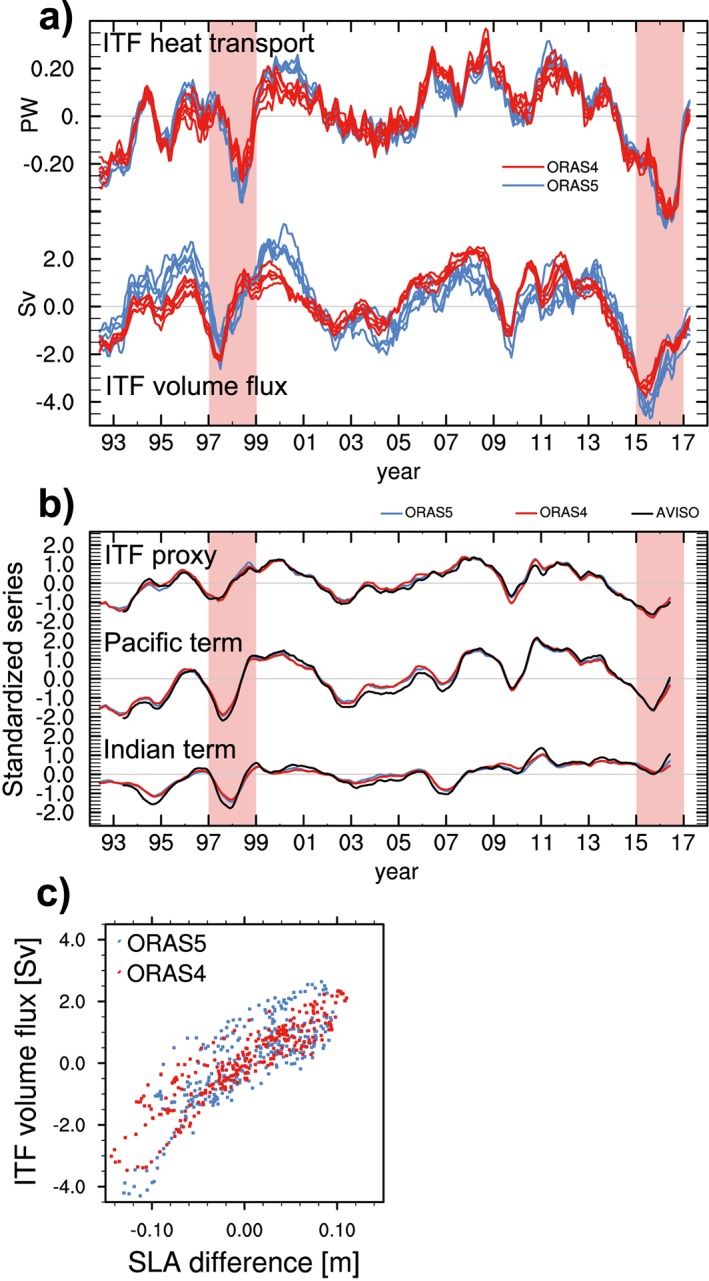
(a) Anomalous Indonesian Throughflow (ITF) (top) heat (in PW; 1 PW = 10^15^ W) and (bottom) volume transports (in Sv; 1 Sv = 10^6^ m^3^ s^−1^) from Ocean Reanalysis System 4 (ORAS4) and Ocean Reanalysis System 5 (ORAS5) (based on data extended to October 2017); (b) standardized anomalies of (top) ITF volume flux proxy and its decomposition into (middle) Pacific and (bottom) Indian Ocean sea level anomaly (SLA) contribution (ITF proxy = Pacific term minus Indian term); based on AVISO, ORAS4, and ORAS5 (ensemble means); and (c) scatter plot of interbasin SLA difference (*x* axis) against reanalyzed ITF volume transport anomalies (positive westward; shown on *y* axis), based on ORAS4 and ORAS5 (ensemble means). The vertical red bars in (a) and (b) indicate the 1997/1998 and 2015/2016 El Niño events, respectively.

Persistent positive ITF heat transports anomalies were observed during 2006–2014 (Hu & Sprintall, 2017; Figure 2a top), which can be linked to the persistent negative state of the Pacific Decadal Oscillation (PDO; Newman et al., 2016) during that period (Ummenhofer et al., 2017). Consistent with these enhanced ITF heat transports, a rapid increase of tropical Indian OHC during that time has been documented (Lee et al., 2015; Nieves et al., 2015), which was much stronger than Pacific warming during 2006–2014 (see supporting information Figure S8).

In contrast to the preceding period, we find a strong reduction in ITF heat transports during 2015/2016, which amounted to −25% over the 2‐year period, unprecedented at least since 1975 (when the ORAS5 record began), both in magnitude and duration (Figure 2a and supporting information Figure S9a). The ITF heat transport reduction during 2015/2016 was led by an exceptional reduction in ITF volume transport (Figure 2a bottom and supporting information Figure S9b). In agreement with conservation of mass, this reduction was balanced by reduced volume transports across the tropical Pacific's southern boundary at 30°S, but this had only a small impact on heat transports across that latitude (see supporting information Figure S7c). It took until early 2017 for the ITF to recover to neutral anomalies (Figure 2a). Note that ITF heat transport anomalies generally lag volume flux anomalies (Figure 2a). This is because velocity anomalies in deeper layers of the ITF generally lead upper layer anomalies (Sprintall et al., 2009; Sprintall & Révelard, 2014), but the latter are more effective in changing heat transports due to warmer water temperatures.

ITF volume fluxes from ORAS4 and ORAS5 agree very well with independent estimates (see supporting information Text and Figure S3); hence, the found anomalies in 2015/2016 are deemed credible. Nevertheless, to further corroborate the results based on the reanalyses and to elucidate the dynamics at play, we estimate ITF volume flux using a dynamic approach based on the interbasin SLA difference between the western equatorial Pacific and the eastern Indian Ocean southwest of Sumatra (Shinoda et al., 2012; regions specified in section 2.2; Figure 2b). Correlation between reanalyzed volume fluxes and the proxy is high (*r* = 0.77 with *p* < 10^−4^ for ORAS5; *r* = 0.92 with *p* < 10^−5^ for ORAS4; see also Figure 2c), indicating that the SLA gradient between the Indo‐Pacific basins contains useful information to explain the strong ITF volume flux reduction. The proxy captures the different magnitude of ITF anomalies during 1997/1998 and 2015/2016, respectively. Decomposition of the proxy shows that SLA in the western Pacific was similar during 1997/1998 and 2015/2016, but the eastern Indian Ocean SLA behaved differently during the two events, apparently explaining the discrepancy in the proxy‐based ITF responses. Around 2007, there is a shift toward prevailing positive SLA in the Indian basin (Figure 2b), consistent with the pronounced increase of Indian OHC from 2006 onward (see supporting information Figure S8). The subsequent Indian basin SLA reduction during the 2015/2016 El Niño was modest compared to 1997/1998, consistent with the fact that the positive Indian Ocean Dipole (IOD; Saji et al., 1999) event was weak in 2015/2016 (L. Liu et al., 2017). It is noteworthy that the relationship between the proxy and ITF volume fluxes shows some indication of nonlinearity (especially in ORAS5; see Figure 2c), with excessive reduction of ITF volume flux when eastern Indian Ocean SLA is high enough.

## 5 Remarkable Changes in Radiation

Tropical Pacific average net radiation anomalies at TOA were positive during the 2015/2016 period (Figure 1c), in contrast to the general picture of radiative heat loss to space during an El Niño event (Loeb et al., 2012; Mayer et al., 2014). To investigate this further, we decompose two‐yearly averaged fields of net radiation anomalies and its decomposition into ASR and OLR during 1997/1998 and 2015/2016.

**Figure 3:**
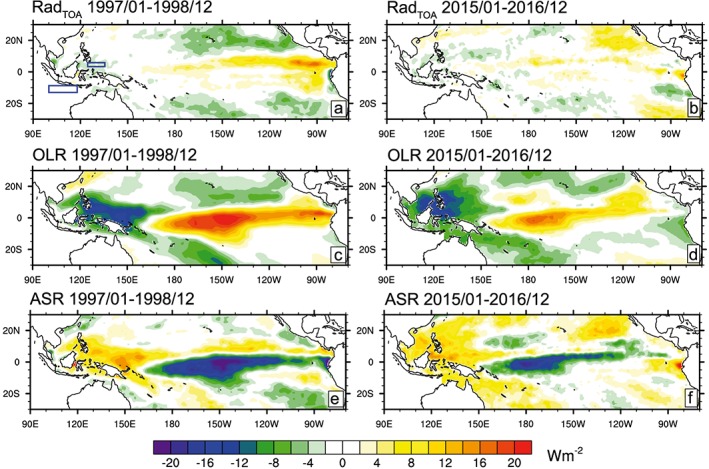
Top‐of‐the‐atmosphere (TOA) radiative energy fluxes (positive down) for 1997–1998 (left column) and 2015–2016 (right column): Net radiation (top); outgoing longwave radiation (OLR) (middle) and absorbed solar radiation (ASR) (bottom). Regions used for computation of the Indonesian Throughflow volume flux proxy are indicated in (a).

Two‐yearly averaged Rad_TOA_ anomalies during 1997/1998 depict pronounced net radiative energy loss in the subtropics (Figure 3a), which is driven by stronger than normal OLR (Figure 3c). The patterns of OLR (Figure 3c) and ASR (Figure 3e) in the Intertropical Convergence Zone (ITCZ) region during 1997/1998 are consistent with anomalous deep convection over the region and largely cancel each other (Kiehl, 1994).

The picture in the ITCZ region is qualitatively similar for OLR (Figure 3d) and ASR (Figure 3f) during 2015/2016, although there is a clear indication of the positive OLR and negative ASR anomalies along the ITCZ extending less far to the east, consistent with less extensive SST anomalies. There is also a pronounced positive ASR anomaly in the northeastern subtropics and to a smaller degree in the southeastern subtropics during 2015/2016, which was not present at all during 1997/1998. These large‐scale anomalies outweighed subtropical OLR anomalies and resulted in positive net radiation anomalies in the eastern subtropical Pacific (Figure 3b) and the surprising result of net radiative energy gain at TOA during 2015/2016.

The explanation for the subtropical ASR anomalies can be found in the persistent positive SST anomalies prevailing since 2014 especially in the northern subtropical Pacific (see supporting information Figure S10). The SST anomalies acted to reduce low‐level cloud cover in that region (see also supporting information Figure S10), resulting in reduced reflected solar radiation (Clement et al., 2009). Whether the pronounced SST anomalies are solely remnants of the weak 2014/2015 El Niño event (Hu & Fedorov, 2016) or are associated also with global warming remains an open question here. Nevertheless, we conclude that it was the unusually warm subtropical SSTs during 2015/2016 that damped surface energy loss of the tropical Pacific.

## 6 Discussion and Conclusion

The results presented here reveal large differences between the energetics of the strong 1997/1998 and 2015/2016 El Niño events. The typical surface flux‐driven cooling of the tropical Pacific associated with El Niño during 2015/2016 was weaker than usual due to enhanced shortwave absorption associated with anomalously low subtropical cloud cover and eventually offset by an exceptional reduction of the ITF heat transport.

We conclude from the presented evidence that the extreme ITF reduction during 2015/2016 had the following precursors: First, the strongly negative PDO during 2006–2014 and associated enhanced Pacific trade winds and ITF heat transports (Lee et al., 2015; Nieves et al., 2015) led to OHC and sea level rise over the Indian Ocean, thereby reducing the mean SLA difference between Indian Ocean and western tropical Pacific.

Second, the very warm eastern Indian Ocean was associated with an anomalously deep thermocline in 2015. Because of this, enhanced Indian Ocean easterly winds as typically associated with El Niño (Annamalai et al., 2003) could not act to cool eastern Indian Ocean SSTs, which prevented subsequent development of a strong positive IOD event via a Bjerknes‐like feedback (Wang et al., 2016). Absence of this feedback in the case of a weakly stratified Indian Ocean with an anomalously deep thermocline has also been found in a climate model (Song et al., 2007). Atmospheric teleconnections from the central Pacific probably played an additional role in weakening the development of the 2016 IOD event (L. Liu et al., 2017).

Third, the absence of a strong positive IOD event during 2015/2016 helped to keep SLA in the eastern Indian Ocean relatively high, contributing to the strong reduction of the ITF. Following this line of arguments, the strong ITF anomaly during 2015/2016 can be viewed as a dynamical rebound effect, as a consequence of the strong Indian Ocean warming in recent years. Thus, consistent with earlier work (e.g., Yamagata et al., 1996), the sea level gradient between eastern Indian Ocean and the western Pacific acts as a flow regulator. This seems to have been the case during 2015/2016.

The absent tropical Pacific heat loss impeded a swing toward longer‐lasting La Niña conditions following the 2015/2016 El Niño event. The tropical Pacific thus remained in a recharged state, characteristic of the positive phase of the PDO. The conditions following the El Niño event were similar to early 2015, being susceptible for forcing wind anomalies to trigger warming events. This result puts in context recent events like the unexpected recurrence of a strong hurricane season in the eastern Pacific during 2016 (Diamond & Schreck, 2017) or the “coastal El Niño” along the Peruvian coast in March 2017 (Fraser, 2017), which are usually not expected after strong El Niño events.

This study shows that energy budget diagnostics of the full tropical Pacific represent a valuable complement to equator‐based standard metrics like thermocline depth or warm water volume. The latter metric tends to be used as a predictor of ENSO and is useful for depiction of equatorial ocean dynamics but is not sufficient to describe the net energetic effect of ENSO. The tropical Pacific OHC could be used as a discerning index to characterize energetics of big ENSO events. We also propose to use the cross‐basin SLA gradient index to monitor and predict the regulating effect of the ITF heat transports on ENSO. Further in‐depth studies including models are needed to further characterize this mechanism.

Earlier studies have found that decadal variability of the Indian Ocean is driven externally, for example, the PDO. Our findings indicate that decadal variability of the Indian Ocean background state can act to modulate interannual variability associated with ENSO. A better understanding of the feedback mechanisms is needed to exploit its potential impact on ENSO predictability.

## Supporting information



Supporting Information S1

Data Set S1

Data Set S2

Data Set S3

Data Set S4

Data Set S5

Data Set S6

Data Set S7

Data Set S8

Data Set S9

Data Set 10
