# Supplementary material for: Unprecedented 2015/2016 Indo‐Pacific Heat Transfer Speeds Up Tropical Pacific Heat Recharge
Source: Geophys Res Lett. 2018 Apr 6;45(7):3274–84. doi: 10.1002/2018GL077106 (PMC5993239; doi:10.1002/2018GL077106)
Supplement: Supplementary file 1 — Supporting Information S1 [file GRL-45-3274-s001.pdf]

AGU PUBLICATIONS

Geophysical Research Letters

Supporting Information for

Unprecedented 2015/16 Indo-Pacific heat transfer speeds up Tropical Pacific heat recharge

Michael Mayer[1,2,*], Magdalena Alonso Balmaseda[2], Leopold Haimberger[1]

[1]Department of Meteorology and Geophysics, University of Vienna, Vienna, Austria.

[2]European Centre for Medium-Range Weather Forecasts, Reading, UK

Contents of this file

Text S1
Text S2
Text and Figure S3
Text and Table S4
Text and Figure S5
Figure S6
Figure S7
Figure S8
Figure S9
Figure S10

Additional Supporting Information (Files uploaded separately)

Captions for Datasets Ds01-Ds10

Text S1. Temporal homogeneity of radiation data

The employed radiation data from University of Reading rely on satellite-based energy-balanced and filled Clouds and the Earth's Radiant Energy system (CERES-EBAF) data from 2000/03 onwards and are assumed to be stable in time, globally and locally. In the early period prior to 2000, the global mean Rad_{TOA} data is also stable (Allan et al. 2014), but we found a discontinuity for the tropical Pacific averages at the time of transition to CERES. We employed a Standard Homogeneity Test using Rad_{TOA} data from the ERA-20CM reanalysis (Hersbach et al. 2015) as a reference, which is assumed to be a stable data set because it uses prescribed SST and does not assimilate any upper-air data. The test indicated a breakpoint exactly between 2000/02 and 2000/03 and the break size for tropical Pacific area-averages of Rad_{TOA} and OLR were estimated to be -1.4 Wm^{-2} and -0.7 Wm^{-2}, respectively. The data prior to 2000/03 were adjusted accordingly.

Text S2. Changes from ORAP5 to ORAS5

ORAS5 uses the same assimilation method and same resolution as ORAP5, but some components have been improved, including use of more up-to-date observational data sets. ORAS5 uses the recently released quality controlled EN4 (Good et al. 2013) in-situ dataset with better vertical resolution and extended coverage than the previous version EN3 used in ORAP5. The altimeter sea-level data has also been updated to use the latest version (DUACS2014) from AVISO. The SST product before 2008 has also been changed and in ORAS5 is based on the Met Office Hadley Centre sea ice and sea surface temperature data set, version 2 (Titchner and Rayner 2014).

Text S3. Validation of ITF transports

As opposed to salinity and temperature, there is no assimilation of ocean currents in ORAS4 and ORAS5. Hence it is necessary to validate the reanalysis-based ITF volume flux against observations. Average ITF volume transport estimates based on ORAS4 and ORAS5 were compared to 2004-2006 mean transport estimates from the International Nusantara Stratification and Transport (INSTANT; Sprintall et al. 2004) campaign. Mean volume transport as estimated from the INSTANT moorings is 15 Sv with a large uncertainty range between 10.7 and 18.8 Sv (Sprintall et al. 2009). ORAS5-based estimates are on the upper end (18.6±0.2Sv) of this range and ORAS4-based estimates lie close to the observational best estimate (14.1±0.2Sv).

It is difficult to validate interannual variability with the three years of INSTANT data, but there exist independent estimates of geostrophic transports based on repeated expendable bathythermograph (XBT) deployments along the IX1 section (Liu et al. 2015). This estimate is most reliable for the upper 400m, where most of the volume flux variability actually occurs (Ming Feng, personal communication 2017). Hence, we compare ORAS4 and ORAS5 upper 400m volume transport anomalies across this section to the estimate from Liu et al. (2015) for the period 1987-2013 (see Figure S3). The agreement on amplitude and phasing of interannual variations is very good for both reanalyses (correlation coefficients around 0.8 for all ensemble members). Since ITF volume flux anomalies also strongly modulate ITF heat transports anomalies, this comparison also provides confidence in our reanalysis-based ITF heat transport estimates.

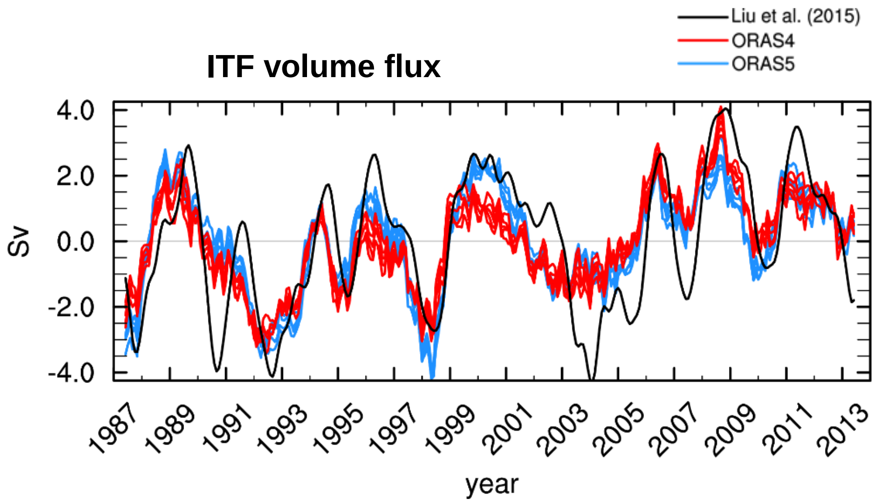

Figure S3. Anomalies of ITF volume flux anomalies based on ORAS4, ORAS5, and Liu et al. (2015). A twelve-month running mean has been applied.

Text S4. Detailed results of forced budget closure

A priori and a posterior estimates and uncertainties for 1997/98 and 2015/16 accumulated terms are provided in table S4. Signs are chosen such that a positive (negative) flux contributes to warming (cooling) of the tropical Pacific.

The budget residual for the 1997/98 event is only -1.8ZJ or 4.4% of the sum of the absolute flux values. For the budget of this event, the largest adjustment is made to OHCT below 300m, where the spread among the different data sets is large, which is due to the very limited observational capabilities in the deeper ocean at that time. The budget residual for the 2015/16 event is 7.0ZJ or 12.7% of the total amount of exchanged energy. The larger imbalance when compared to 1997/98 is surprising since the ocean was much better observed in recent years, as reflected by the lower uncertainty estimates for OHCT, especially below 300m. However, the spread among the atmospheric reanalyses, on which AHT is based, was relatively large. One reason for this might be the switch to operational SSTs in ERA-Interim after 2009 (Dee et al. 2011), probably introducing a temporal discontinuity. We note that for the budgets of both event, all adjustments are smaller than one standard deviation of the respective uncertainty estimates.

	1997/98				2015/16			
	F_i'	σ_i'	F_i	σ_i	F_i'	σ_i'	F_i	σ_i
Rad_{TOA}	-2.1	1.5	-2.1	1.5	3.9	1.0	4.1	1.0
AHT	-9.3	1.2	-9.3	1.2	-13.1	4.8	-8.9	3.1
AET	-0.3	0.1	-0.3	0.1	0.1	0.1	0.1	0.1
ITF	7.2	2.1	7.2	2.0	18.0	1.0	18.2	1.0
OHT_{30N}	-1.2	0.6	-1.2	0.6	2.0	0.9	2.1	0.9
OHT_{30S}	-6.8	0.8	-6.8	0.8	-1.5	1.5	-1.0	1.5
$OHCT_{0-300m}$	-11.7	3.0	-11.5	2.8	10.1	1.8	9.6	1.7
$OHCT'_{300m-full}$	-2.5	9.7	-1.0	3.9	6.4	2.8	5.1	2.5
residual	-1.8	-	0	-	7.0	-	0	-

Table S4. Unadjusted (Fi') and adjusted (F_i) energy budget terms (accumulated anomalies) including uncertainty estimates based on the methods and datasets described earlier in this section. Signs are chosen such that positive (negative) fluxes contribute to a warming (cooling) of the tropical Pacific. Units are ZJ.

Text S5. Under assumption of Sverdrup balance, temporarily accumulated zonal mean wind stress curl can be used as a proxy for meridional warm water export from the equator. Fig. S5 shows temporally accumulated wind stress curl in the Pacific between 5N and 5S during 1997/98 and 2015/16. Since the anomalous meridional warm water export from the tropical Pacific is computed as the difference of anomalous northward transports across 5N and 5S, respectively, the presented quantity actually represents the difference of wind stress curl area-averaged between 0-5N and 0-5S. The curves start do diverge in boreal spring 1998 and 2016, following the respective peaks of the El Ninos. This is exactly when the OHC curves in Fig.1a of the main text start to diverge. This means that anomalous wind stress curl lasted for much longer during the 97/98 event. This is consistent with the fact that during the decay phase in 1998 warm SST anomalies in the eastern equatorial Pacific persisted a few months longer than in 2016 (Paek et al. 2017), which helped to keep up the wind (curl) anomalies and heat discharge for several more months.

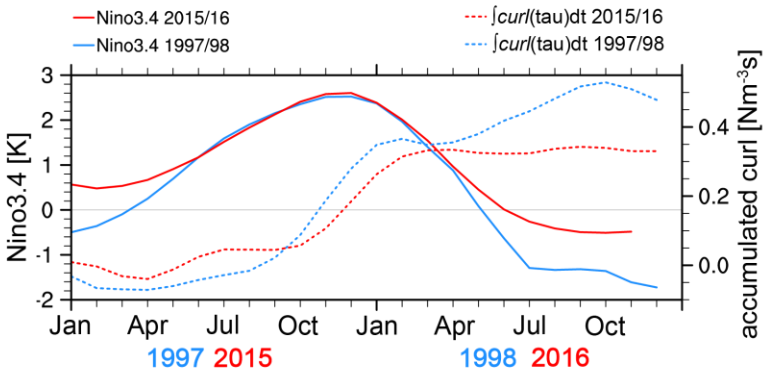

Figure S5: Nino 3.4 (SST averaged over 5S-5N and 170-120W) anomalies [K] and accumulated wind stress curl anomalies [N m^{-3} s] along the equatorial Pacific (right axis) for 1997/98 and 2015/16 (both based on ERA-Interim data).

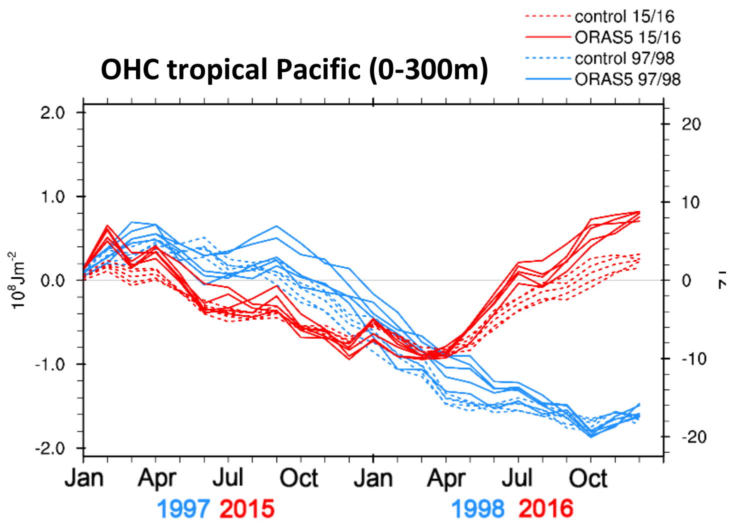

Figure S6. Tropical Pacific OHC (0-300m) evolution for 1997/01-1998/12 and 2015/01-2016/12 based on data from ORAS4 and a control run (forced model run without data assimilation) with otherwise same configuration as ORAS5.

Figure S7. Accumulated heat transport anomalies for the three oceanic boundaries of the tropical Pacific. Values are with respect to tropical Pacific total area and signs are chosen that positive values contribute to a warming of the tropical Pacific.

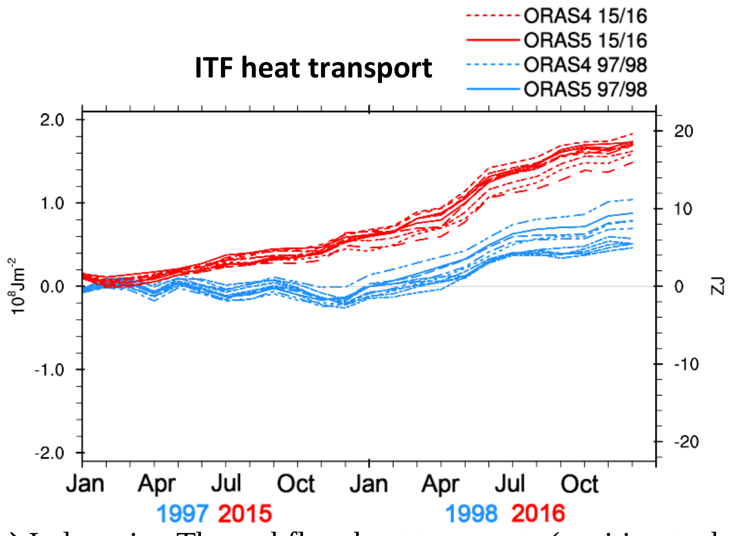

a) Indonesian Throughflow heat transports (positive to the East).

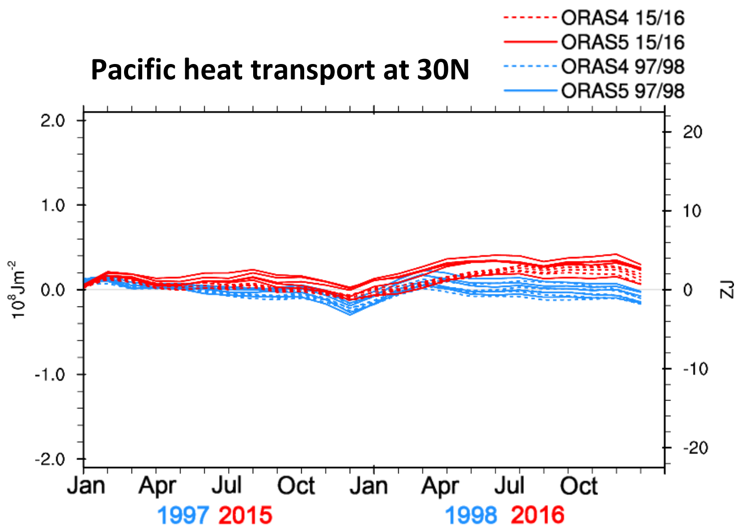

b) Pacific ocean heat transports across 30N (positive to the South).

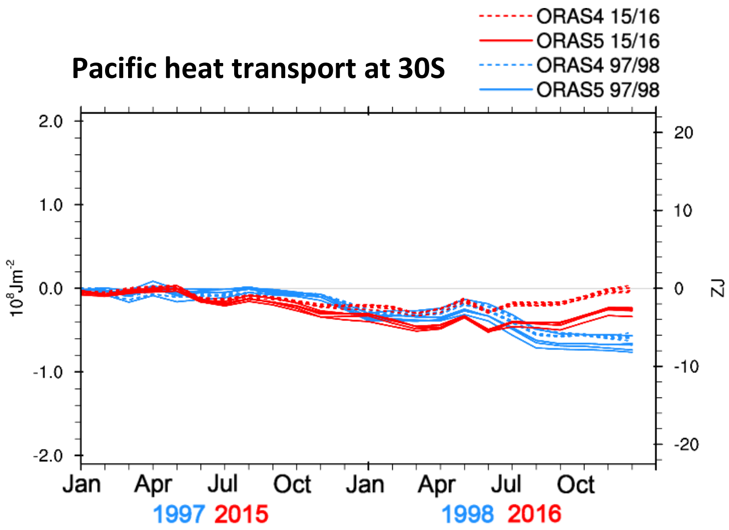

c) Pacific ocean heat transports across 30S (positive to the North).

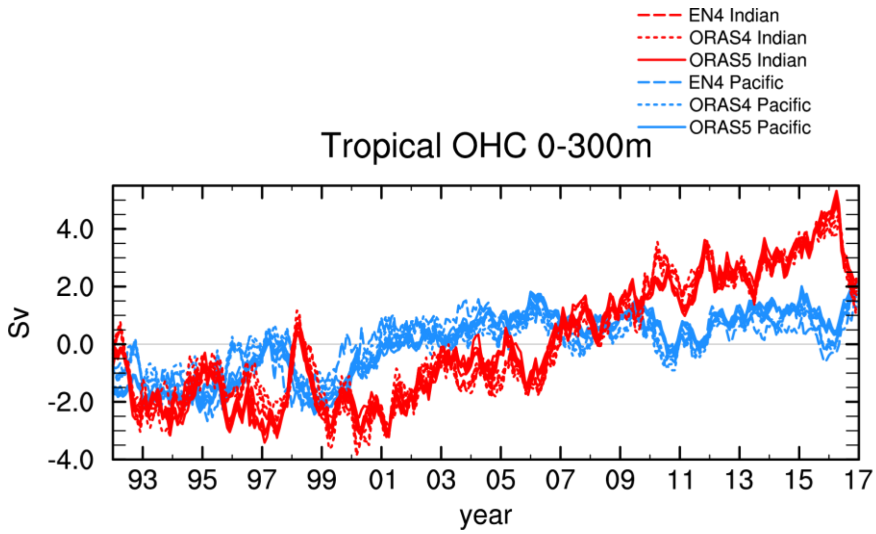

Figure S8. Tropical Indian and Pacific Ocean upper OHC (0-300m; 30S-30N) anomalies [10^8 Jm-2]. Note that a change of 10^8 Jm^{-2} corresponds to a column-averaged temperature change of about 0.083 K.

a)

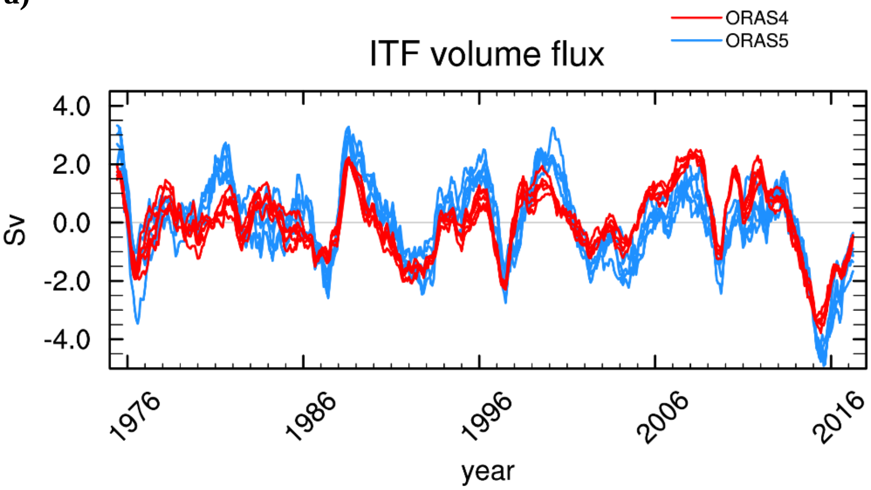

b)

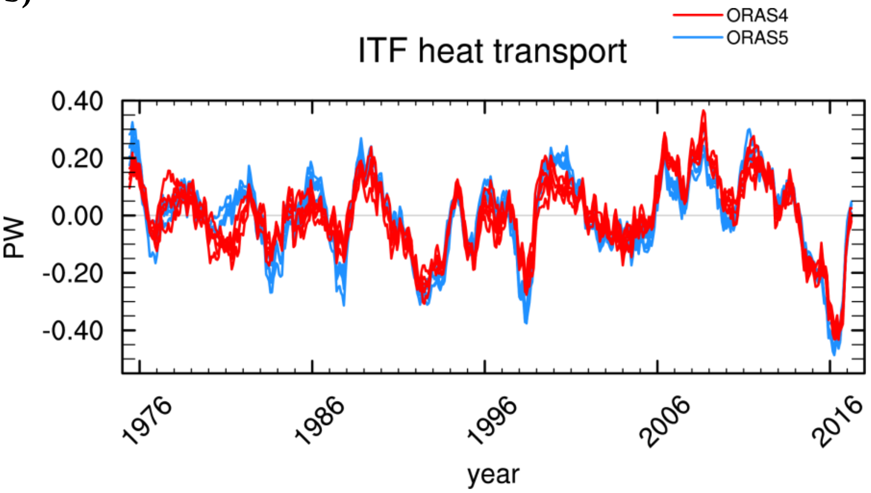

Figure S9. Anomalous ITF a) heat transports and b) volume flux from ORAS4 and ORAS5 (based on data extended to October 2017), beginning in 1975 (the first year of ORAS5)

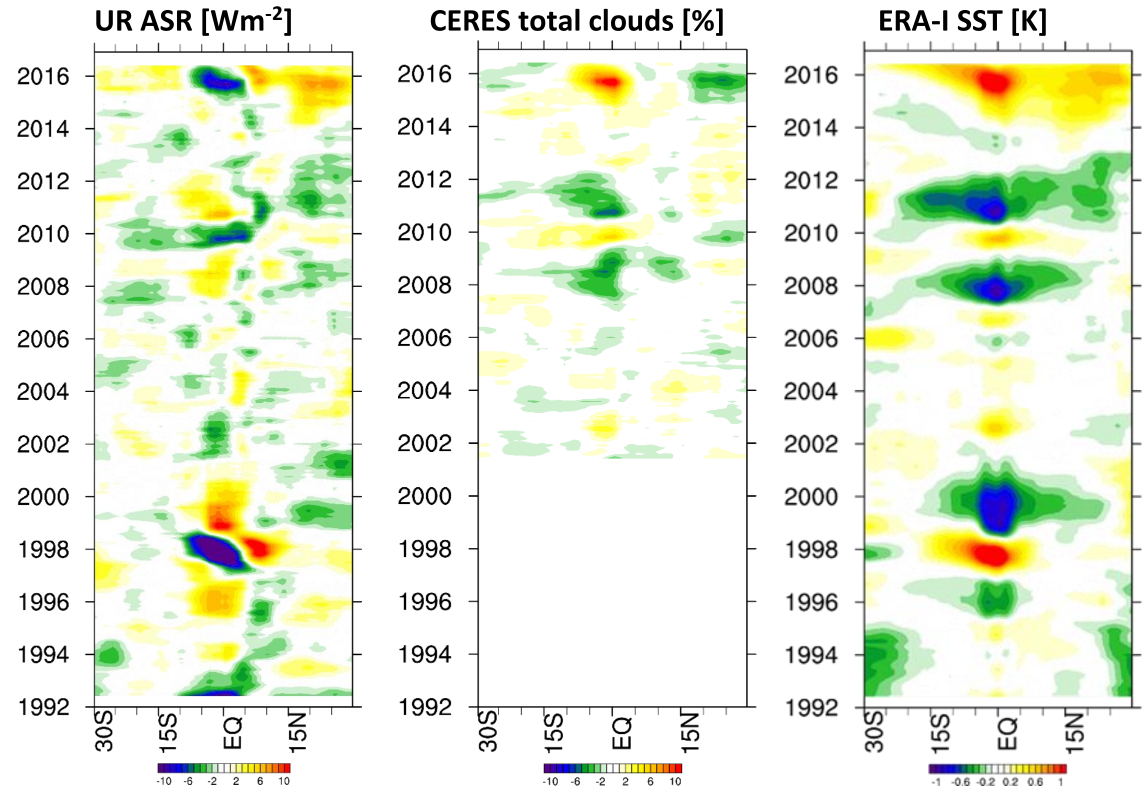

Figure S10. Pacific zonal average University of Reading (UR) absorbed solar radiation (left; Wm^{-2}), satellite-based total cloud coverage (middle; percentage) and ERA-Interim SST (right; K). Clouds are shown for illustration of the relationship between SST anomalies and ASR anomalies via clouds. However, neither CERES nor UR provide temporally homogeneous cloud data prior to 2000/03.

Dataset Ds01. ORAS4 Pacific 30N full-depth heat transport in PW (5 members beginning in 1975/01)

Dataset Ds02. ORAS4 Pacific 30S full-depth heat transport in PW (5 members beginning in 1975/01)

Dataset Ds03. ORAS4 ITF full-depth heat transport in PW (5 members beginning in 1975/01)

Dataset Ds04. ORAS4 ITF full-depth volume transport in Sv (5 members beginning in 1975/01)

Dataset Ds05. ORAS5 ITF 0-400m heat transport in Sv (5 members beginning in 1975/01)

Dataset Ds06. ORAS5 Pacific 30N full-depth heat transport in PW (5 members beginning in 1975/01)

Dataset Ds07. ORAS5 Pacific 30S full-depth heat transport in PW (5 members beginning in 1975/01)

Dataset Ds08. ORAS5 ITF full-depth heat transport in PW (5 members beginning in 1975/01)

Dataset Ds09. ORAS5 ITF full-depth volume transport in Sv (5 members beginning in 1975/01)

Dataset Ds10. ORAS5 ITF 0-400m heat transport in Sv (5 members beginning in 1975/01)
